# Characterization of the Tyrosine Kinase-Regulated Proteome in Breast Cancer by Combined use of RNA interference (RNAi) and Stable Isotope Labeling with Amino Acids in Cell Culture (SILAC) Quantitative Proteomics*[Fn FN2]

**DOI:** 10.1074/mcp.M115.048090

**Published:** 2015-06-18

**Authors:** Justin Stebbing, Hua Zhang, Yichen Xu, Arnhild Grothey, Paul Ajuh, Nicos Angelopoulos, Georgios Giamas

**Affiliations:** From the ‡Department of Surgery and Cancer, Division of Cancer, Imperial College London, Hammersmith Hospital Campus, Du Cane Road, London, W12 ONN, UK;; §Dundee Cell Products Ltd, James Lindsay Place, Dundee Technopole, Dundee, DD1 5JJ, UK

## Abstract

Tyrosine kinases (TKs) are central regulators in cellular activities and perturbations of TK signaling contribute to oncogenesis. However, less than half of the TKs have been thoroughly studied and a global functional analysis of their proteomic portrait is lacking. Here we conducted a combined approach of RNA interference (RNAi) and stable isotope labeling with amino acids in cell culture (SILAC)-based quantitative proteomics to decode the TK-regulated proteome and associated signaling dynamics. As a result, a broad proteomic repertoire modulated by TKs was revealed, upon silencing of the 65 TKs expressed in MCF7 breast cancer cells. This yielded 10 new distinctive TK clusters according to similarity in TK-regulated proteome, each characterized by a unique signaling signature in contrast to previous classifications. We provide functional analyses and identify critical pathways for each cluster based on their common downstream targets. Analysis of different breast cancer subtypes showed distinct correlations of each cluster with clinical outcome. From the significantly up- and down-regulated proteins, we identified a number of markers of drug sensitivity and resistance. These data supports the role of TKs in regulating major aspects of cellular activity, but also reveals redundancy in signaling, explaining why kinase inhibitors alone often fail to achieve their clinical aims. The TK-SILACepedia provides a comprehensive resource for studying the global function of TKs in cancer.

Cancer is a heterogeneous disease harboring aberrant genomic alterations (1). Although extensive transcriptome and epigenomic analyses have provided us with data at the genetic level and their translation to specific phenotypes, a proteomic portrait and mapping of signaling pathways in cancer is potentially more insightful and accurate, albeit technologically more challenging (2). Recently, mass spectrometry (MS)-based quantitative proteomics have been widely applied in cancer research, allowing large scale, robust, and confident identification of biochemical networks implicated in cancer (3).

Stable isotope labeling with amino acids in cell culture (SILAC)^1^-coupled MS analysis represents one of the most promising comparative quantitative methods that has been broadly employed in proteomic research generating vast amounts of functional data (4–8). This approach enables clear identification and quantification of protein dynamics essential in oncogenesis and therefore has been extensive utilized in cancer-proteome studies (9, 10). For instance, a global proteomic analysis acquired by SILAC-based MS has provided information about the protein changes related to breast tumor progression as well as novel prognostic markers (11). A comprehensive phosphoproteome analysis has also been applied to identify and quantify the dynamics in signaling networks upon epidermal growth factor (EGF) stimulus delineating an integrative picture of cellular regulation (5). SILAC mouse coupled to high-resolution MS was employed to measure the phosphoproteomic alterations in skin cancer development (12). Recently, the application of SILAC-based quantitative proteomics allowed a detailed assessment of surface proteome between interphase and mitosis in human cancer cells, providing potential pharmacodynamic biomarkers for antimitotic cancer chemotherapy (10). Furthermore, loss-of-function studies using genome-wide RNAi screens are a powerful tool and have been successfully performed to identify molecular targets as well as key regulators in biologic processes in cancer (13–15). Therefore, combined use of RNAi and SILAC-based quantitative proteomics would be a potentially powerful approach for dissecting cellular signaling in tumorigenesis.

The involvement of tyrosine kinases (TKs) in basic cellular processes and their contribution to development and progression of diseases has been widely described (16). TK protein abundance as well as alterations in the total and/or phosphorylated levels of proteins encompassed in TK signaling pathways can contribute to tumorigenesis (16). Given their involvement in cancer, targeted therapy against TKs has been one aspect of therapy that has undoubtedly improved the clinical outcome of patients in the past decade. However, resistance to these treatments inevitably develops, largely because of the aberrant activation of other TKs possessing a complementary or compensatory function (17). Furthermore, less than half of the TKs described thus far have been thoroughly studied and even in these cases a global functional analysis and understanding of their proteomic portrait is lacking (18).

Here, for the first time we describe the global mapping of TK-regulated proteome using a high throughput RNAi screen combined with SILAC-based quantitative proteomics in MCF7 breast cancer cells. Overall, four thousand distinct proteins were detected and quantified in the TK-silencing data sets showing a diverse landscape of modulated proteins. Based on the similarity in their proteomic changes, we present 10 new distinctive clusters from the 65 TKs and ultimately characterize a unique proteomic signature and functional portrait of each cluster. These data suggest that despite the primary structural homology of kinases, their regulated proteome can vary significantly and may depend on other factors, notably their dynamic interactions with other proteins. We also present clinical associations of each cluster with survival data in patients with different molecular breast cancer subtypes. In addition, based on the downstream effects of the identified TK-clusters, we identify genes that are most significantly associated with sensitivity/resistance to doxorubicin. Our defined functional analysis of the TK-regulated proteome supports a fundamental involvement of TKs in the major cellular processes and highlights a compensatory activation in their associated signaling. In addition to the analyses presented herein, our established database present a unique resource that can be utilized for comparative and integrative analyses, for inspection of function of each TK of interest, and their associated signaling networks in cancer.

## EXPERIMENTAL PROCEDURES

### 

#### 

##### RNA Extraction and RT-qPCR

RNeasy kit (Qiagen, Manchester, UK) was used to isolate total RNA. Reverse transcription was performed using high capacity cDNA reverse transcription kit (Applied Biosystems, Warrington, UK). RT-qPCR analysis was performed on a 7900HT Thermocycler (Applied Biosystems) using SYBR^®^ Green master mix and primers for the whole tyrosine kinase family, purchased from Qiagen.

##### SILAC Cell Culture

To generate SILAC conditions, normal DMEM medium deficient in arginine (R) and lysine (K) was supplemented with stable isotope-encoded arginine and lysine as previously described (19, 20). For “medium” labeling we used l-[^13^C_6_] arginine (R6) and l-[^2^H_4_] lysine (K4), and for “heavy” labeling we used l-[^13^C_6_, ^15^N_4_] arginine (R10) and l-[^13^C_6_, ^15^N_2_] lysine (K8). For the “light” condition, or unlabeled, l-[^12^C_6_,^14^N_4_] arginine (R0), and l-[^12^C_6_,^14^N_2_] lysine (K0) were used. Final concentrations of arginine were 84 mg/L and 146 mg/L of lysine. The tissue culture media (Dundee Cell Products, Dundee, UK) were supplemented with 10% dialyzed fetal bovine serum (FCS) with 10kDa MWCO, 1% (10 mg/ml) streptomycin/(10,000 units/ml) penicillin, 2 mm glutamine, and 1 mm sodium pyruvate. MCF7 cells that were grown for at least seven passages prior to this experiment.

##### Western Blotting

RIPA buffer (Sigma, Hertfordshire, UK) added with fresh protease and phosphatase inhibitor was used to extract whole cell lysates. Cell pallets were mixed thoroughly with RIPA lysis buffer, and then incubated in ice for 15 min before centrifuging at 15,000 rpm for 15 min at 4 °C. Protein concentration was measured by the bicinchoninic acid (BCA) protein assay (Pierce, Paisley, UK). Lysates were heated with 5× sodium dodecyl sulfates (SDS) sample buffer at 95 °C for 5 min before they were loaded to 10% SDS-PAGE. Samples were then transferred to Hybond ECL super nitrocellulose membranes (GE Healthcare). Subsequently the membranes were blocked in TBS containing 0.1% (v/v) Tween20 and 5% (w/v) nonfat milk for 1 h. The primary antibodies were probed with membranes overnight at 4 °C. The membranes were then washed three times in TBS/Tween for 15 min following incubation with HRP-conjugated secondary antibodies (1:3000 dilution) for 60 min. The membranes were then washed three times again and were detected with enhanced chemiluminescence (ECL). Films were developed using a Konica SRX-1001A x-ray developer. Alternatively, membranes were incubated with IRDye® Donkey anti-mouse or Donkey anti-rabbit secondary antibodies for 60 min and visualized by Odyssey® Fc Imaging System (LI-COR®).

##### RNAi Screening in MCF7 Cells

The human Tyrosine Kinase family siRNAs from Qiagen was used. The library was supplied in a 96-well format (Qiagen FlexiPlate) and contained a pool of two individual verified siRNAs per well, targeting two different sequences for each gene. The knockdown efficiencies of all siRNAs were validated by RT-qPCR and Western blotting. For RNAi screening, MCF7 cells were grown in SILAC medium as described above for at least seven passages and then seeded into 100 mm dishes. Subsequently, cells were transfected with either siControl or verified siRNAs against the tyrosine kinases (final concentration 40 nm) and HiPerFect reagent according to the manufacturer's instructions (Qiagen) for 72 h in indicated SILAC medium, followed by proteomic analysis.

##### Protein Digestion and Peptide Fractionation

Equal amounts of protein from unlabeled and labeled samples were combined prior to protein digestion. Briefly, samples were reduced in 10 mm dithiothreitol and alkylated in 50 mm iodoacetamide prior to boiling in loading buffer, and then separated by one-dimensional SDS-PAGE (4–12% Bis-Tris Novex mini-gel, Invitrogen, Paisley, UK) and visualized by colloidal Coomassie staining (Novex, Invitrogen). The entire protein gel lanes were excised and cut into 10 slices each. Every gel slice was subjected to in-gel digestion with trypsin overnight at 37 °C. The resulting tryptic peptides were extracted by formic acid (1%) and acetonitrile (CH_3_CN), lyophilized in a speedvac, and resuspended in 1% formic acid.

##### Mass Spectrometry Methods

Trypsin-digested peptides were separated using an Ultimate 3000 RSLC (Thermo Scientific) nanoflow LC system. On average 0.5 μg was loaded with a constant flow of 5 μl/min onto an Acclaim PepMap100 nanoViper C18 trap column (100 μm inner-diameter, 2 cm; Themro Scientific). After trap enrichment, peptides were eluted onto an Acclaim PepMap RSLC nanoViper, C18 column (75 μm, 15 cm; Thermo Scientific) with a linear gradient of 2–40% solvent B (80% acetonitrile with 0.08% formic acid) over 65 min with a constant flow of 300 nl/min. The HPLC system was coupled to a linear ion trap Orbitrap hybrid mass spectrometer (LTQ-Orbitrap Velos, Thermo Scientific) via a nano electrospray ion source (Thermo Scientific). The spray voltage was set to 1.2 kV, and the temperature of the heated capillary was set to 250 °C. Full-scan MS survey spectra (*m*/*z* 335–1800) in profile mode were acquired in the Orbitrap with a resolution of 60,000 after accumulation of 1,000,000 ions. The fifteen most intense peptide ions from the preview scan in the Orbitrap were fragmented by collision-induced dissociation (normalized collision energy, 35%; activation Q, 0.250; and activation time, 10 ms) in the LTQ Orbitrap after the accumulation of 10,000 ions. Maximal filling times were 1000 ms for the full scans and 150 ms for the MS/MS scans. Precursor ion charge state screening was enabled, and all unassigned charge states as well as singly charged species were rejected. The dynamic exclusion list was restricted to a maximum of 500 entries with a maximum retention period of 180 s and a relative mass window of 15 ppm. The lock mass option was enabled for survey scans to improve mass accuracy. Data were acquired using the Xcalibur software.

##### Proteome Quantification

The raw mass spectrometric data files obtained for each experiment were collated into a single quantitated data set using MaxQuant (1.3.0.5.) (21) and the Andromeda search engine software (1.3.0.5.) (22). The sequence database used was from ipi.HUMAN v.3.68 with 87,061 entries. Enzyme specificity was set to that of trypsin, allowing for cleavage N-terminal to proline residues and between aspartic acid and proline residues. Other parameters used were: (1) variable modifications, methionine oxidation, protein N-acetylation, gln → pyro-glu; (2) fixed modifications, cysteine carbamidomethylation; (3) database: target-decoy human MaxQuant; (4) heavy labels: R6K4 and R10K8; (5) MS/MS tolerance: FTMS- 10ppm, ITMS- 0.6 Da; (6) maximum peptide length, 6; (7) maximum missed cleavages, 2; (8) maximum of labeled amino acids, 3; and (9) false discovery rate, 1%. Peptide ratios were calculated for each arginine- and/or lysine-containing peptide as the peak area of labeled arginine/lysine divided by the peak area of nonlabeled arginine/lysine for each single-scan mass spectrum. Peptide ratios for all arginine- and lysine-containing peptides sequenced for each protein were averaged. Data are normalized using 1/median ratio value for each identified protein group per labeled sample. The mass spectrometry proteomics data have been deposited to the ProteomeXchange Consortium (23) via the PRIDE partner repository with the data set identifier PXD002065. Annotated spectra for all results can be accessed using MS-Viewer (24) via the links provided from supplemental Table S5.

##### Bioinformatics and Statistical Analyses

The bioinformatic analyses were performed in R (25) and SWI-Prolog (26), using Real (27) for connecting the two systems. Specifically, the hierarchical clustering of SILAC proteomics data after silencing TKs was performed based on calculated distances by R's hclust function. The complete linkage method that aims to find similar clusters based on overall cluster measure was used. For the correlation heatmap, we calculated and plotted pairwise distances between tyrosine kinases according to their protein quantification signatures. The distance metric was conducted using the centered Pearson correlation.

To visualize the TK-modulated proteomics, the heatmap of quantified values showing the overall pattern of regulation was displayed. Individual proteins were plotted in either red for down-regulated proteins, or white for nondifferential and nonidentified, or blue for up-regulated proteins. Significance B test was performed to characterize the most significantly regulated proteins after silencing of TKs (*p* < 0.05) (21).

For each identified cluster, GO analysis was performed at the level 2 of the three GO domains: biologic process (BP), cellular component (CC), and molecular function (MF) (28). Furthermore, the hyper-geometric test from GOstats package was used to identify GO terms that are enriched in the deregulated genes in each cluster (29). For every overrepresented GO term, a network connecting deregulated genes in this GO term was drawn showing the networks among these genes in the STRING protein–protein interactions database (30). Only edges with a confidence value greater than 500 were included (range: 0–999). SWI-Prolog was used for the overall control of our pipeline and the graph operations on the STRING database.

To investigate the effect of our clusters to doxorubicin sensitivity in a panel of breast cancer cell lines, we integrated data from the cancer cell line encyclopedia (CCLE) (31) and drug-response signature from the genomic markers screening of drug sensitivity in cancer (GDSC) (32). From the downstream effects (up- or down-regulated proteins) of each cluster, expression values from CCLE for these genes were regressed against the GDSC IC_50_ values for doxorubicin for breast cancer cell lines (37 cell lines, linear regression with ANOVA <0.01).

##### Kaplan-Meier (KM) Plotter Analysis

KM Plotter online survival analysis, which has massive gene expression data and survival information of more than 3000 patients, was used (33). Gene expression data and relapse free and overall survival information are publically available from the Gene Expression Omnibus (GEO, http://www.ncbi.nlm.nih.gov/geo/) and The Cancer Genome Atlas (TCGA). The database is handled by a PostgreSQL server, which integrates gene expression and clinical data simultaneously. To analyze the prognostic value of a particular gene, the patient samples are split into two groups according to quantile expressions of the proposed biomarker. In this study, the mean expression levels of various TKs in each classified cluster were selected. All the 65 identified TKs were examined except EPHA6 in cluster 2 and LMTK3 in cluster 6 because of lack of data. The relevance on relapse free survival (RFS) in luminal A, luminal B, and basal-like breast cancer was assessed. A survival curve with the hazard ratio (HR) with 95% confidence intervals and logrank *p* value is displayed.

## RESULTS

### 

#### 

##### Expression Profile and Silencing of the TK Family in MCF7 Cells

We initially examined the expression profile of all members of the TK family in the MCF7 breast cancer cell line. A quantitative RT-qPCR analysis was performed to determine the endogenous expression levels of all 90 TKs including 58 receptor tyrosine kinases (RTKs) and 32 cytoplasmic tyrosine kinases (CTKs). A cut-off of threshold cycle (CT) value was set at <34.5, above which the data have a high chance of resulting from cross contamination or amplification of fluorescent artifacts (using GAPDH as endogenous control housekeeping gene with a CT value of ∼17). Based on this analysis, 66 TKs expressed at variable levels with CT values ranging from 21.5 to 34.5 (supplemental Fig. S1*A*) were then individually silenced using a siRNA library composed of two siRNAs/targeted gene, followed by RT-qPCR to verify the knockdown efficiency (RNAi sequences used for each gene are shown in supplemental Table S1). Out of 66 TKs, 65 were successfully silenced with a decrease of ≥70% at the mRNA levels (supplemental Fig. S1*B*). Further validation of the siRNA efficacy was assessed by Western blotting on several TKs (supplemental Fig. S1*C*).

##### Establishing an Approach for Identification of TK Signaling by Combined Use of Quantitative Proteomics SILAC and RNAi Screen

Considering the documented role and involvement of TKs in cancer signaling (16) and the fact that a complete picture of the TK-regulated proteome is still lacking, we performed a SILAC-based quantitative proteomic analysis of the breast cancer tyrosine kinome after siRNA treatment. Prior to application of combined use of SILAC and RNAi, we have validated the high reproducibility, high sensitivity and high accuracy of SILAC-based MS in two independent studies (19, 20).

**Fig. 1.**
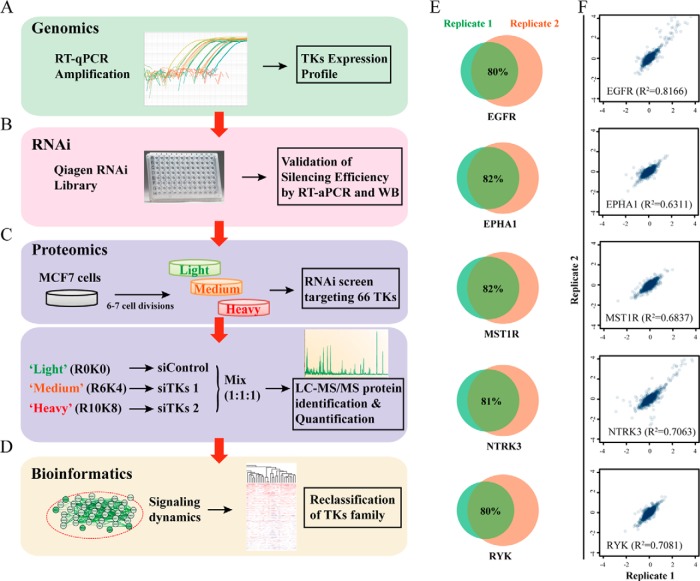
**Strategy for identification of TK signaling by combined use of SILAC-based quantitative proteomics and RNAi.** *A*, The expression profile of all 90 TKs was examined using RT-qPCR in MCF7 cells. *B*, Silencing efficiency of a certified RNAi library composed of two siRNAs per each TK was validated by RT-PCR and Western blotting. *C*, MCF7 cells were then grown in either R0K0 “light” or unlabeled medium l-[12C6,14N4] arginine (R0) and l-[12C6,14N2] lysine (K0), or R6K4 “medium” labeled l-[13C6] arginine (R6) and l-[2H4] lysine (K4), or R10K8 “heavy” medium labeled l-[13C6, 15N4] arginine (R10) and l-[13C6, 15N2] lysine (K8). Subsequently, SILAC-based proteomic analysis was performed after silencing each verified TKs in MCF7 cells. *D*, Further bioinformatic analyses were implemented to reclassify the family of TKs and to characterize the associated functional portrait. *E*, Validation of the experimental approach performed in this study. Venn graphs showing a high overlap of identified proteome between different replicates after silencing EGFR, EPHA2, MST1R, NTRK3, and RYK are presented. *F*, Comparison of quantifications between different replicates after silencing EGFR, EPHA2, MST1R, NTRK3, and RYK. Correlation analysis of the protein ratios plotted against each replicate is shown.

We therefore established an approach incorporating RT-qPCR, RNAi, SILAC-based quantitative proteomics, and bioinformatics analysis to characterize the TKs-regulated proteome (Fig. 1*A*–1*D*). Specifically, the expression profile of all 90 TKs was examined using RT-qPCR in MCF7 cells. Further validation of the silencing efficiency of a certified RNAi library against TKs was done by RT-qPCR and Western blotting. After SILAC-cell labeling (as described under “Experimental Procedures”), R0K0, R6K4, and R10K8 labeled cells were transfected with either control siRNA or a pool of two validated siRNAs targeting two individual TKs, respectively. Subsequent MS analysis was conducted and further bioinformatic analyses were implemented to reclassify the family of TKs and to characterize their associated functional portraits.

Replicates of several randomly selected TKs, including EGFR, EPHA2, MST1R, NTRK3, and RYK were performed following the same protocol. Approximately 80% of the proteome acquired in different replicates was shown identical, highlighting a high degree of overlap between replicates (Fig. 1*E*). Importantly, the quantifications between replicates were greatly consistent shown by correlation analysis of the protein ratios plotted against each replicate (Fig. 1*F*). Moreover, the expressions of differentially regulated proteins upon silencing of individual TKs were selectively validated using Western blotting, further confirming the reproducibility of our data (supplemental Fig. S2).

##### TKs Silencing Induces Global Proteomic Alterations in MCF7 Cells

For each individual TK, from ∼27,000 nonredundant peptide sequences, a SILAC-based proteome was assembled comprising more than 2000 distinguishable and unambiguously identified proteins with a minimum of two peptides with a false discovery rate (FDR) of 1% (minimal peptide length of seven amino acids). The mass spectrometry proteomics data have been deposited to the ProteomeXchange Consortium (23) via the PRIDE partner repository (accession: PXD002065). Detailed information for each identified protein, including protein IDs, number of peptides used for identification, and % coverage is also shown in supplemental Table S2. From these proteins, more than 2000 were quantified and their distribution according to fold changes was determined (supplemental Fig. S3 and the key features of the SILAC analyses are summarized in supplemental Table S3).

**Fig. 2.**
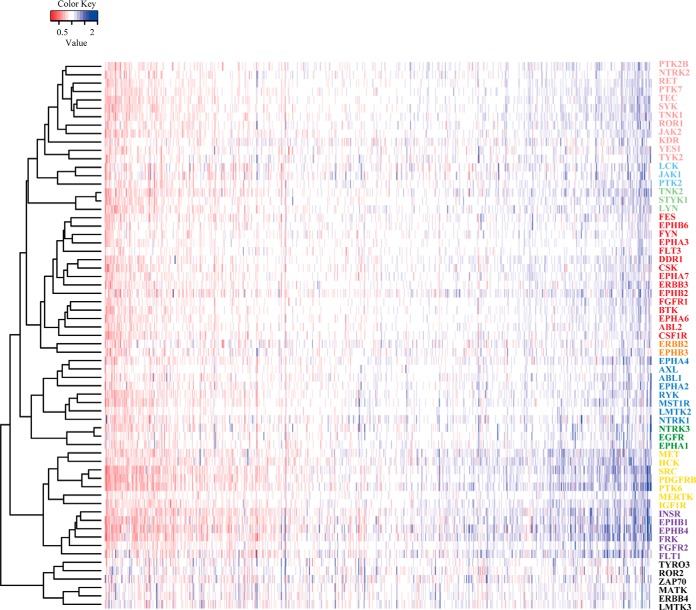
**Heatmap of quantified proteins after TK silencing.** The overall pattern of regulation is shown in the heatmap of quantified values. After normalized to siControl, values of fold changes are all above zero, with value one showing that the expression levels of the specific protein are not altered after silencing TKs. For each knockdown (rows) the quantified value for an identified protein is plotted in red for down regulated proteins (below one), white for nondifferential and nonidentified and blue for up-regulated proteins (above one). The row labels indicate the knock out experiment and the colors correspond to the clusters described below.

A SILAC ratio representing the fold change of protein abundance after individually silencing 65 TKs was compared and normalized to control group (siControl). After normalization, values of fold changes are all above 0, with value 1 (0 on the log_2_ scale) showing that the expression levels of the specific proteins are not altered after TKs silencing. To acquire a portrait of the TK-regulated proteome, the overall pattern of regulation is shown in the heatmap of quantified values. To improve resolution and concentrate the plot in the most significantly enriched region, ∼1000 identified proteins were presented. For each knockdown (rows), the quantified values are plotted in red for down-regulated, white for no-change and nonidentified and blue for up-regulated proteins (Fig. 2). Furthermore, the SILAC proteomic quantifications also confirmed the efficient knockdown of the individual TKs and a quantification heatmap of cross identification of TKs in all experiments are displayed (supplemental Fig. S4). In some cases, the silenced TKs proteins were not identified, because of either innate low abundance or high silencing efficiency, which did not allow the detection (*i.e.* EGFR, EPHA1, EPHB2, EPHB3, EPHB4, EPHB6, ERBB2, ERBB3, JAK1, PTK2B, PTK7, and YES1). Other strongly down-regulated TKs that were detected during their silencing include CSK, DDR1, IGF1R, PTK2, and RET.

##### Cluster Identification of the TKs in MCF7 Cells

**Fig. 3.**
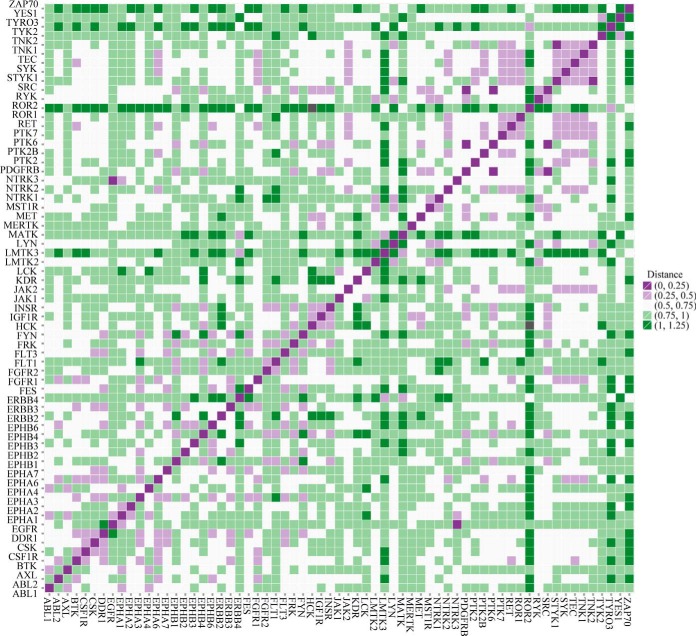
**Correlation heatmap of the distance metric between all 65 TKs in MCF7 cells.** Pairwise distances were calculated and plotted according to the quantifications of TK-regulated proteomic signatures using centered Pearson correlation. In the correlation heatmap showing the distance metric between all kinases in our study, the smaller distances are displayed in purple, whereas the longer distances are in green.

**Fig. 4.**
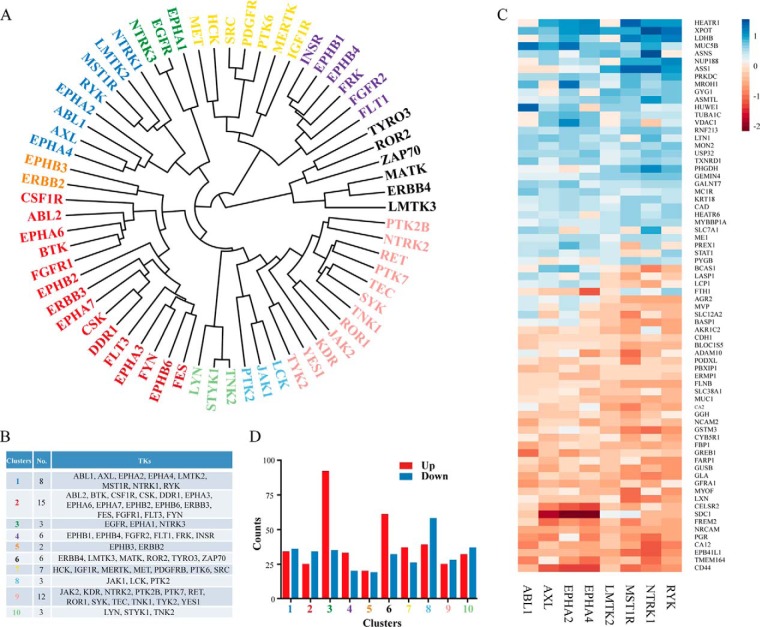
**Hierarchical clustering of the 65 TKs expressed in MCF7 cells.** *A*, Hierarchical clustering of the 65 TKs was performed using R's hclust function. The complete linkage method which aims to identify similar clusters based on overall cluster measure was used. Ten distinctive clusters were obtained and the complete dendrogram is shown with the labels colored for these clusters. *B*, Full list of the TKs included in each cluster. The color-coding of the clusters is used throughout to identify the analysis relevant to the corresponding clusters. *C*, Heatmap of the proteomic quantifications (log_2_ values of normalized fold changes against control) for the downstream effects (significantly up- or down-regulated proteins, Significant B test *p* < 0.05) after silencing TKs in cluster 1. *D*, Number of proteins significantly up or down-regulated in each identified cluster. *x* axis shows 10 different clusters and *y* axis indicates the counts.

We then calculated and plotted pairwise distances based on the quantifications of TK-regulated proteomic signatures using centered Pearson correlation (27). In the correlation heatmap showing the distance metric between all kinases in our study, the smaller distances are displayed in purple corresponding to similar signatures, whereas the longer distances are in green (Fig. 3). Ten distinctive clusters were obtained, according to the similarity of each TK-regulated proteome and the complete dendrogram is shown with the labels colored for these clusters (Fig. 4*A*). The number and a full list of TKs in each cluster are presented and the color coding of the clusters is used throughout to identify the analysis relevant to the corresponding clusters (Fig. 4*B*).

##### Characterization of a Proteomic Signature and Functional Portrait for Each TK Cluster

We next investigated the TK-regulated proteomic changes within each classified cluster in more detail to delineate the proteomic signature of each cluster. A decent proportion of proteins showed significant quantitative change after knockdown of TKs in each cluster (*p* < 0.05 according to Significance B calculation) (21). A full list of proteins that are significantly regulated in each cluster is presented in supplemental Table S4. To characterize the top hits within each cluster, we firstly filtered the proteins by the requirement that they should be identified in at least 70% of the TKs in a cluster. We then selected the proteins that have a Significant B value of *p* < 0.05 in at least half the cluster members. A heatmap showing the log_2_ ratio values of hit proteins was generated for each cluster and is shown in Fig. 4*C* and supplemental Fig. S5. Counts of the most significantly regulated proteins in each cluster are shown in Fig. 4*D*. All the subsequent analyses were conducted based on these significant hits.

**Fig. 5.**
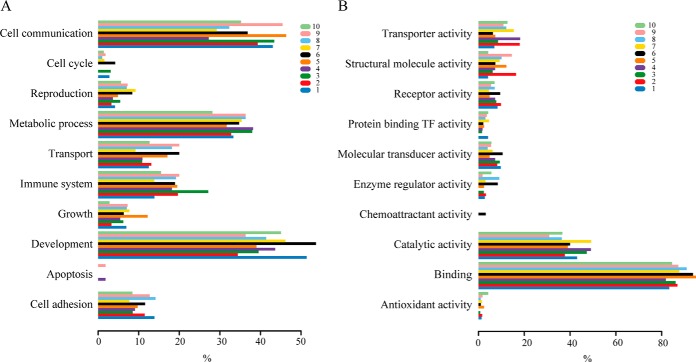
**Characterization of a functional portrait for each cluster.** *A*, A functional profile of top GO biologic processes that the up- and down-regulated proteins belong to is presented. *x* axis shows the percentage of hits in each cluster that belong to a GO biologic process term. The color coding and the number for each cluster are indicated as above. *B*, A functional profile of top GO molecular functions that the up- and down-regulated proteins belong to is presented. *x* axis shows the percentage of hits in each cluster that belong to a GO molecular function term.

To gain insights into the biologic processes distinctively modulated by each cluster, we identified the top GO categories to which the differential proteins in each cluster belong. We ran the GO analysis on cellular components (CC), biologic processes (BP) and molecular function (MF). Overall, the identified proteins are scattered in a variety of cellular components, including extracellular matrix, cell membrane, cytoplasm, nuclei, synapse, and other organelles. They are involved in a wide range of cellular activities such as immune system process, reproduction, metabolic process, growth, cell communication, development, cell cycle, transcription, and apoptosis, whose deregulation can contribute to oncogenesis (Fig. 5*A*).

**Fig. 6.**
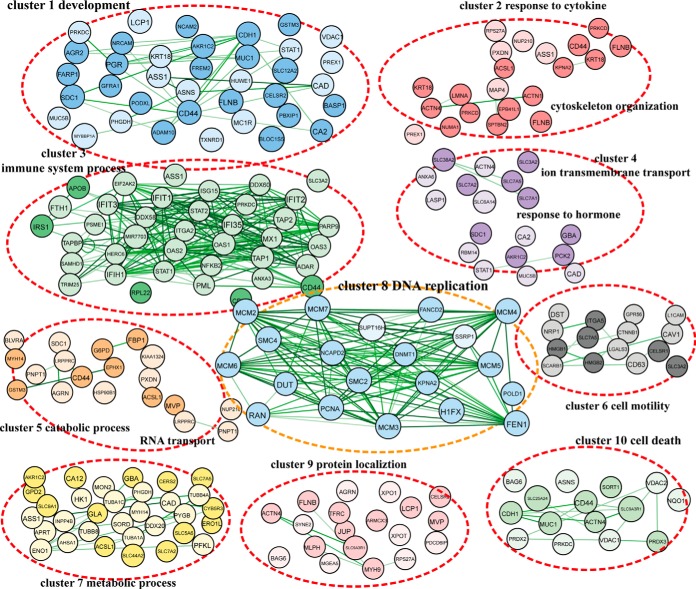
**Representatives of defined functional networks in each classified TK cluster.** The functional networks were generated using GO analysis combined with the STRING platform. Proteins in lighter color are up-regulated, whereas brighter color indicates down-regulation. Arrows show the interactions between connected proteins. Representative defined functional networks associated with their clusters are shown here. The color coding and the number for each cluster are indicated as above.

The GO analysis of molecular functions showed a large percentage of proteins with binding, catalytic, structural, and enzyme regulatory activities within each cluster (Fig. 5*B*). This supports the established role of TKs as important modulators in signal transduction via functional and physical protein–protein interactions. In addition, the comparative abundance of genes provides a high-level functional signature for the clusters. We thus sought to integrate the GO analyses with the STRING database to delineate the enrichment of functional protein–protein interaction networks in each cluster (30). First, we identified GO terms that were over-represented in the differential proteins of each cluster. We then depicted the STRING network for the differential proteins within an overrepresented GO term. Color-coded networks were generated to distinguish up- (lighter) or down-regulation (brighter) of proteins. As expected, comprehensively integrated subnetworks emerged in a functional linked manner. The subnetworks from each cluster uncovered an exclusive feature of functional connections contributed by the regulated proteome and also highlighted the complexities of TK-modulated cellular signaling. Representatives of the most enriched functional networks within each cluster are displayed (Fig. 6): in cluster 1, development; in cluster 2, response to cytokine; in cluster 3, immune response; in cluster 4, response to hormone; in cluster 5, catabolic process and RNA transport; in cluster 6, cell motility; in cluster 7, metabolic process; in cluster 8, DNA replication; in cluster 9, protein localization; in cluster 10, cell death.

Instead of structural homology, our classification highlights similarity in biologic function and signaling networks shared among TKs in different clusters. Significantly, this clustering verifies current knowledge of the recognized functions of TKs, and also indicates the potential involvement of TKs in undocumented biologic processes, which merits further investigation. For example, the most significantly enriched functional network related to the kinases in cluster 6 (ERBB4, LMTK3, MATK, ROR2, TYRO3, ZAP70) is cell motility. We have recently shown that lemur receptor tyrosine kinase 3 (LMTK3) promotes invasion in breast cancer through GRB2-mediated induction of integrin family members, supporting our data presented here (19, 34). Another good example is manifested in cluster 8, which comprises JAK1, LCK, and PTK2. In this study, the bioinformatics analysis revealed a key characteristic of cluster 8 that is its contribution to DNA replication initiation and elongation during cell cycle through regulating minichromosome maintenance (MCM) proteins (MCM2–7) (Fig. 6), whose deregulation is associated with genomic instability and cancer (35–38). Our SILAC proteomic data showed that, upon silencing of the three kinases in cluster 8, protein levels of all MCM members were decreased, which would subsequently lead to an inhibition in uncontrolled DNA replication in cancer. Ultimately, silencing TKs can block sustained replicative immortality of tumor cells. This finding is consistent with the current perceptions of TKs, being oncogenic therapeutic targets in cancer.

##### Utilization of the TKs Proteomic Repertoire for Designated Analyses

To show a broader significance of our proteomic database in studying the downstream effects and associated signaling modulated by TKs, two representative example analyses were performed. Firstly, to investigate the effect of knockdown of TKs belonging to the same cluster on common transcription factors (TFs), we utilized a repertoire of 1391 TFs previously identified (39). A total of 221 TFs were quantified in at least one SILAC knockdown experiment in our data set and 73 common TFs were quantified in a minimum of 55 knockdowns. We clustered the TFs according to their values in our experiments upon individual TK knockdown. We then plotted the SILAC proteomic measurements of these TFs against the TKs (supplemental Fig. S6). Importantly, the clusters identified by the proteomic data indicate new functional relationships among transcription factors. For instance, the regulation pattern of different TFs, such as LARP1, CUL2, and TCF25, by the TKs is consistently observed in the data set, highlighting a potential similarity in their biological function. Furthermore, we also saw consistent patterns of coregulation of TFs by different cluster members. For example, cluster 6 (black) shows a strong pattern throughout the whole TFs, whereas there is a marked up-regulation of SSRP, UBTF, and PREX1 in cluster 2 (red). The new grouping of TFs shows a broad regulation pattern of TFs by different kinases in the same cluster, suggesting various cross-talks between TFs and TKs, as well as between members within each family.

We also present another example exploiting the effect of TKs knockdowns on proteins involved in translation initiation. We then mapped the overall regulation of eukaryotic translation initiation factors (EIFs) by TKs in our data set. Forty-two EIFs were quantified and a hierarchical grouping of the EIFs family was generated (supplemental Fig. S7). Moreover, our analysis revealed interesting functional connections among EIFs modulated by TKs. For instance, EIF4A1, EIF4B, and EIF3I were affected in a similar way by the majority of the TKs, although they have not been linked functionally before. Our results indicate an essential role of TKs in regulating the initiation of protein synthesis, but the related biological interpretation requires further investigation.

##### The Clinical Significance of Each Classified TK Cluster in Different Molecular Subtypes of Breast Cancer

**Fig. 7.**
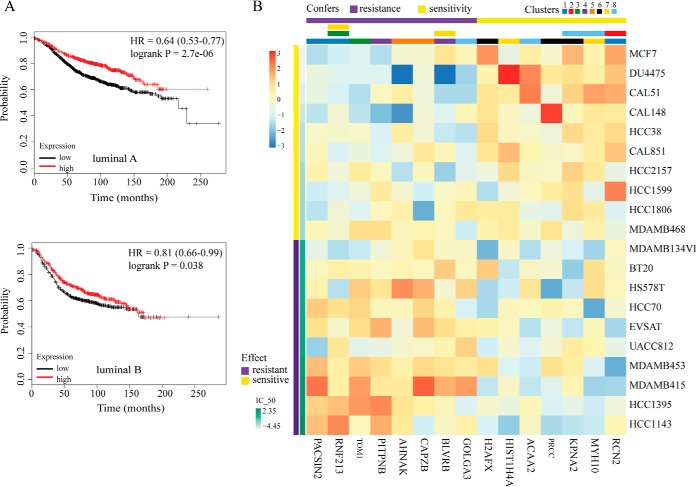
**Associations of each classified TK with clinical outcomes and drug response in breast cancer.** *A*, Clinical significance of classified TK clusters in different molecular subtypes of breast cancer. Kaplan-Meier curves showing associations of expression levels of TKs in cluster 1 with relapse free survival (RFS) in luminal A and luminal B breast cancer were presented. *B*, Heatmap for the associations of each classified TK cluster with drug sensitivity and resistance in breast cancer cell lines. Gene expressions of downstream effects (up- and down-regulated) in each cluster were gained from CCLE and regressed against the GDSC IC50 values for doxorubicin in breast cancer cell lines. The genes those were most significantly associated with sensitivity (yellow)/resistance (purple) to doxorubicin are presented here (linear regression with ANOVA *p* < 0.01). The color coding and the number for each cluster are indicated as above.

Breast cancer is a heterogeneous disease with certain subtypes having better outcomes than others (40). The main molecular subtypes defined by gene expression profiling include luminal A, luminal B, basal-like, HER2-enriched, and normal-like (41–43). To further uncover the association of gene expression levels of our identified clusters with clinical outcome in different subtypes, we utilized the online survival analysis tool KM Plotter, which has massive gene expression data and survival information derived from more than 3000 patients from databases including The Cancer Genome Atlas (TCGA) (33). Considering that the MCF7 cell line belongs to the luminal subtype, the relevance of the mean expression levels of various TKs in each classified cluster on relapse free survival (RFS) in luminal A and luminal B breast cancer was assessed (Fig. 7*A* and supplemental Fig. S8). Our results showed a distinctive correlation for each cluster with survival time in different subtypes of breast cancer. For example, in cluster 1, the higher average expression levels of ABL1, AXL, EPHA2, EPHA4, LMTK2, MST1R, NTRK1, and RYK associated with a longer RFS in luminal A and luminal B patients, whereas the lower average expression levels of cluster 5 (EPHB3 and ERBB2) or cluster 10 (LYN, STYK1, and TNK2) genes correlated with a longer RFS in luminal B and luminal A subtypes respectively. This further confirms that breast cancer represents a diverse spectrum of diseases with distinct biologic features and varied clinical outcome. In many cases, predictive or prognostic significance to survival cannot be simply attributed to single markers. Conversely, our identified TK clusters revealing complex molecular signatures and biologic functions might serve as complementary panel biomarkers to the known established ones, such as ERα and PgR, to have prognostic and therapeutic implications.

##### Profile of Each Classified TK Cluster for Drug Sensitivity and Resistance in Breast Cancer Cell Lines

Aberrant regulations of genes or proteins (including mutations, amplification, and overexpression) are known to contribute to sensitivity or resistance of cancer cells to therapeutic drugs (32, 44). To address whether our established TK clusters could be used to predict the therapeutic effectiveness in response to drugs in breast cancer, we integrated the transcriptome data representing the mRNA expression from the CCLE (31) and doxorubicin-response signature from the GDSC (32). We initially sought to identify genes correlated to sensitivity/resistance to doxorubicin and then established the TK clusters significantly regulating these genes.

Expression values from CCLE for the genes that are most differentiated in each cluster in our data set were regressed against the GDSC IC_50_ values for doxorubicin for a panel of breast cancer cell lines (37 cell lines, linear regression with ANOVA *p* < 0.01). In aggregate, our analysis revealed a total of 15 genes that are significantly associated with sensitivity/resistance to doxorubicin, which belong to eight TK clusters (Fig. 7*B*). In some instances, the identified genes have already been shown a potential value for assessing response to cancer treatment, such as neuroblast differentiation-associated protein (AHNAK) and H2A histone family, member X (H2AX). For example, two recent studies have reported a significant gene-drug correlation between AHNAK expression and chemotherapy compounds, including paclitaxel, docetaxel, erlotinib, everolimus, and doxorubicin in breast cancer (45, 46). In addition, several lines of evidence have supported an important role of γH2AX as a biomarker of drug effect in solid tumors, including breast and lung cancer (47–49).

On the other hand, some genes identified above have not yet been related with drug resistance in cancer. For instance, protein kinase C and casein kinase substrate in neurons (PACSIN2) has been documented as a regulator in cell spreading and migration through interacting with Rac1 and an important modulator in EGFR internalization (50, 51). Ring finger protein 213 (RNF213) has been reported as a fusion partner of ALK and MYC in anaplastic large cell lymphoma and inflammatory myofibroblastic tumor (52, 53).

Of note, as shown in Fig. 7*B*, our results showed that multiple genes from clusters 3 and 5 confer resistance whereas genes from clusters 2 and 6 primarily confer sensitivity. Furthermore, our analysis showed that numerous identified genes belong to more than one cluster (*i.e.* RNF213 is regulated by TK clusters 1, 3, and 7). These findings reveal a link between our clusters with doxorubicin response and highlight again that TKs from different clusters can compensate for each other through governing same downstream targets. Further investigation and validation of these new targets in regulating drug resistance is urgently needed and will advance our understanding in this field.

## DISCUSSION

SILAC coupled to high-resolution MS is shown here and by other studies to be a simple yet powerful method for investigating the dynamics of protein abundance and post-translational modifications (4–8). Here, we describe the global mapping of the TK-regulated proteome and the signaling dynamics using a high throughput RNAi screen combined with SILAC-based quantitative proteomics in MCF7 cancer cells.

Most early studies have focused on elucidating the function of individual member within the TK family in cancer; different papers are hard to compare because of the use of different cell lines and conditions and in addition, only a few TKs have been thoroughly studied. A global functional analysis of the TK regulated-proteome has been, until now, lacking (18). With recent progress in global quantitative proteomics, this large-scale, robust identification of biochemical networks implicated in cancer is now feasible (3), as we have shown. We believe that a complete proteomic portrait provides new valuable insights at a global level.

A new classification of the TK family was established containing 10 distinctive clusters based on similarity of the regulated proteome, highlighting a functional redundancy between various TKs within specific clusters. This new TK-grouping also offers a possible explanation to the failure of single TK-targeted therapy, as certain members of a specific group may compensate for the down-regulation or loss of other members in the same group. The clustering presented herein differs to the canonical classification of TKs, although in some cases the two classifications are in agreement. As expected, the number of TKs in each of the 10 clusters varies significantly, with 15 in the largest group and only two in the smallest one. This variation might indicate that in the larger families, TKs tend to function cooperatively to regulate signaling pathways, whereas in the smaller groups TKs are more likely to act alone.

Our analyses uncovered numerous significantly modulated proteins, most of which are unique in each cluster. A distinctive signature for regulated proteins in each group was derived. In general, identified proteins appear widely distributed across different cellular compartments and are implicated in multiple biological processes with dysregulation of these contributing to tumorigenesis. Further combined analyses with the STRING database showed a clear picture of integrated functional protein–protein interaction networks modulated by the TK proteome. GO analysis revealed that although each cluster has a unique functional signature, there is also some functional overlap between clusters. This further supports the notion that TKs in different clusters can contribute to the activation of the same pathways and compensate for each other through signaling cross-talk.

Our clustering also suggests the participation of TKs in undocumented biological processes that await further investigation. In one example, functional analysis of the proteome modulated by JAK1, LCK, and PTK2 suggests their involvement in DNA replication through governing the MCM protein family (35–38). Silencing of these kinases in cluster 8 caused reduced protein levels of all detected MCM protein members. This may result in inhibition of sustained replicative immortality of tumor cells, which supports current opinions that TKs are tumorigenic and can represent therapeutic targets in cancer. Moreover, we present two examples analyzing the regulation pattern of the TFs and EIFs family by TKs to show the broader significance of our data set.

Additionally, we reveal a distinct association for each cluster with relapse free survival in different molecular subtypes of breast cancer. It would be intriguing to incorporate our classified clusters with the established biomarkers to add prognostic and predictive information for patients with different subtypes of breast cancer. Finally, we observed interesting links between our TK clusters and a number of genes/proteins implicated in doxorubicin sensitivity.

To fully interpret the data from SILAC proteomics, further *in vitro* and *in vivo* studies are required to investigate the most enriched functional networks in each cluster. It would be of interest to characterize and compare the TK-regulated phospho-proteomic profile using a similar strategy. Notably, global proteomic analysis that can complement genomic studies can help us study cellular regulation such as protein interaction, and can uncover novel mechanisms contributing to tumor progression (12, 54).

In conclusion, this is the first global proteomic signature of TKs in breast cancer and a valuable resource for studying the function of TKs in cancer. Of note, TKs can compensate for each other to maintain functional activity upon disruption and can contribute to the same downstream signaling in tumor initiation and development. We offer an explanation for the observed resistance to TK-targeted inhibitors considering the plasticity and reciprocity between TKs. Our data also reveals a distinct association for each cluster with survival in breast cancer. It emphasizes a rationale for the development of a combination of multitargeted TKs as antitumor drugs. Furthermore, we uncover several biochemical networks that will help us further understand and elucidate the cross-talk between cellular signaling pathways implicated in breast cancer.

## Supplementary Material

Supplemental Data

## Footnotes

^1^ The abbreviations used are:

SILAC: stable isotope labeling with amino acids in cell culture
EGF: epidermal growth factor
TK: tyrosine kinase
CCLE: cancer cell line encyclopedia
GDSC: genomic marker screening of drug sensitivity in cancer
RFS: relapse free survival
RTK: receptor TK
CTK: cytoplasmic TK
CT: cut-off of threshold
EIF: eukaryotic translation initiation factor.
