# Supplementary material for: Characterization of the Tyrosine Kinase-Regulated Proteome in Breast Cancer by Combined use of RNA interference (RNAi) and Stable Isotope Labeling with Amino Acids in Cell Culture (SILAC) Quantitative Proteomics
Source: Mol Cell Proteomics. 2015 Jun 18;14(9):2479–92. doi: 10.1074/mcp.M115.048090 (PMC4563730; doi:10.1074/mcp.M115.048090)
Supplement: Supplemental Data [file supp_14_9_2479__index.html]

Reprogramming of the tyrosine kinase-regulated proteome in breast cancer by combined use of RNAi and SILAC quantitative proteomics — Characterization of the Tyrosine Kinase-Regulated Proteome in Breast Cancer by Combined use of RNA interference (RNAi) and Stable Isotope Labeling with Amino Acids in Cell Culture (SILAC) Quantitative Proteomics — Biomarker Discovery of Bladder Cancer by Tissue Proteomics — Supplemental Data 

# Characterization of the Tyrosine Kinase-Regulated Proteome in Breast Cancer by Combined use of RNA interference (RNAi) and Stable Isotope Labeling with Amino Acids in Cell Culture (SILAC) Quantitative Proteomics

## Supplemental Data

- Supplemental information - Supplemental information
- Supplemental Table 1 - A full list of siRNA sequences used for targeting TKs in this study
- Supplemental Table 2 - Detailed information for each identified protein, including protein IDs, number of peptides used for identification and % coverage
- Supplemental Table 3 - key features of the SILAC-based analysis.
- Supplemental Table 4 - A full list of proteins that are significantly regulated in each cluster
- Supp Table S5 - Supp Table S5
