# Supplementary material for: Characterization of the Tyrosine Kinase-Regulated Proteome in Breast Cancer by Combined use of RNA interference (RNAi) and Stable Isotope Labeling with Amino Acids in Cell Culture (SILAC) Quantitative Proteomics
Source: Mol Cell Proteomics. 2015 Jun 18;14(9):2479–92. doi: 10.1074/mcp.M115.048090 (PMC4563730; doi:10.1074/mcp.M115.048090)
Supplement: Supplemental Data [file supp_M115.048090_mcp.M115.048090-1.pdf]

Reprogramming of the tyrosine kinase-regulated proteome in breast cancer by combined use of RNAi and SILAC quantitative proteomics

Justin Stebbing[1,¥], Hua Zhang[1,¥,*], Yichen Xu[1], Arnhild Grothey[1], Paul Ajuh[2], Nicos Angelopoulos[1] and Georgios Giamas[1*]

[1]Department of Surgery and Cancer, Division of Cancer, Imperial College London, Hammersmith Hospital Campus, Du Cane Road, London, W12 ONN, UK
[2]Dundee Cell Products Ltd, James Lindsay Place, Dundee Technopole, Dundee, DD1 5JJ

Keywords: cancer/ cellular signaling /proteomics/ SILAC/tyrosine kinase

[¥] These Authors contributed equally to this study.

*To whom correspondence should be addressed:

Drs Georgios Giamas and Hua Zhang
Imperial College London
Division of Cancer
Hammersmith Hospital Campus
IRDB Building
Du Cane Road
W12 0NN
London, UK
E mail: g.giamas@imperial.ac.uk or h.zhang10@imperial.ac.uk

Supplemental figure 1

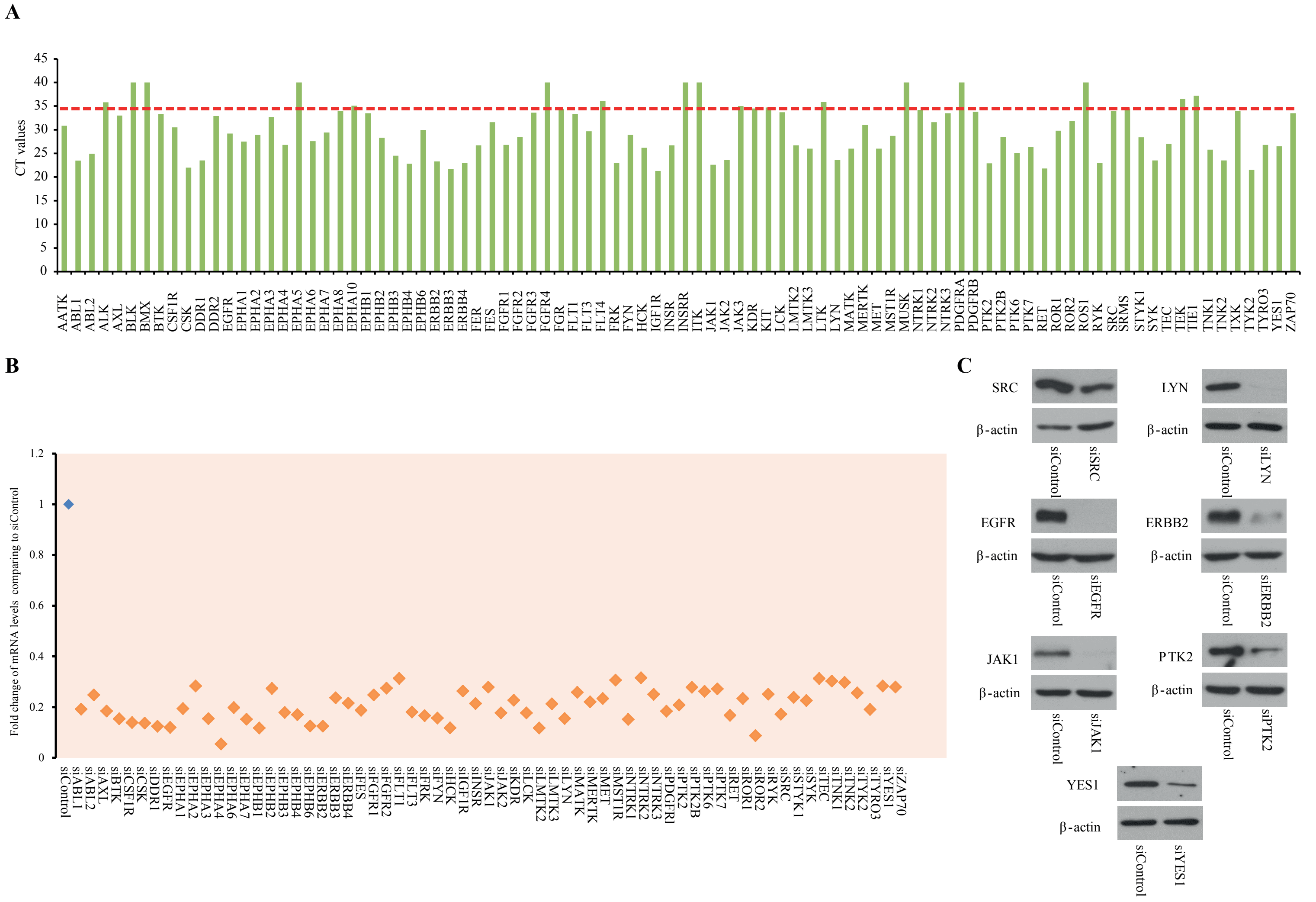

Supplemental Fig. S1. Expression profile of TK family members in MCF7 breast cancer cells and validation of siRNAs-targeting TKs. A, A quantitative RT-qPCR analysis was performed to determine the endogenous expression levels of all 90 TKs in MCF7. A cut-off threshold cycle (CT) value was set as 34.5, above which the data can be interpreted as results of cross contamination or amplification of fluorescent artefacts. 66 TKs are expressed at variable levels with a CT value ranging from 21.5 to 34.5. B, MCF7 cells were transfected with an siRNA library composed of 2 siRNAs/targeted TK gene for 72 hours. RT-qPCR was then performed to verify the knockdown efficiency of the siRNAs. Fold changes of mRNA levels comparing to siControl (blue) are presented here. C, MCF7 cells were transfected with either siControl or siRNAs-targeting TKs for 72 hours. Western blotting was then performed to verify the knockdown efficiency of the siRNAs on 7 randomly chosen TKs as indicated.

Supplemental figure 2

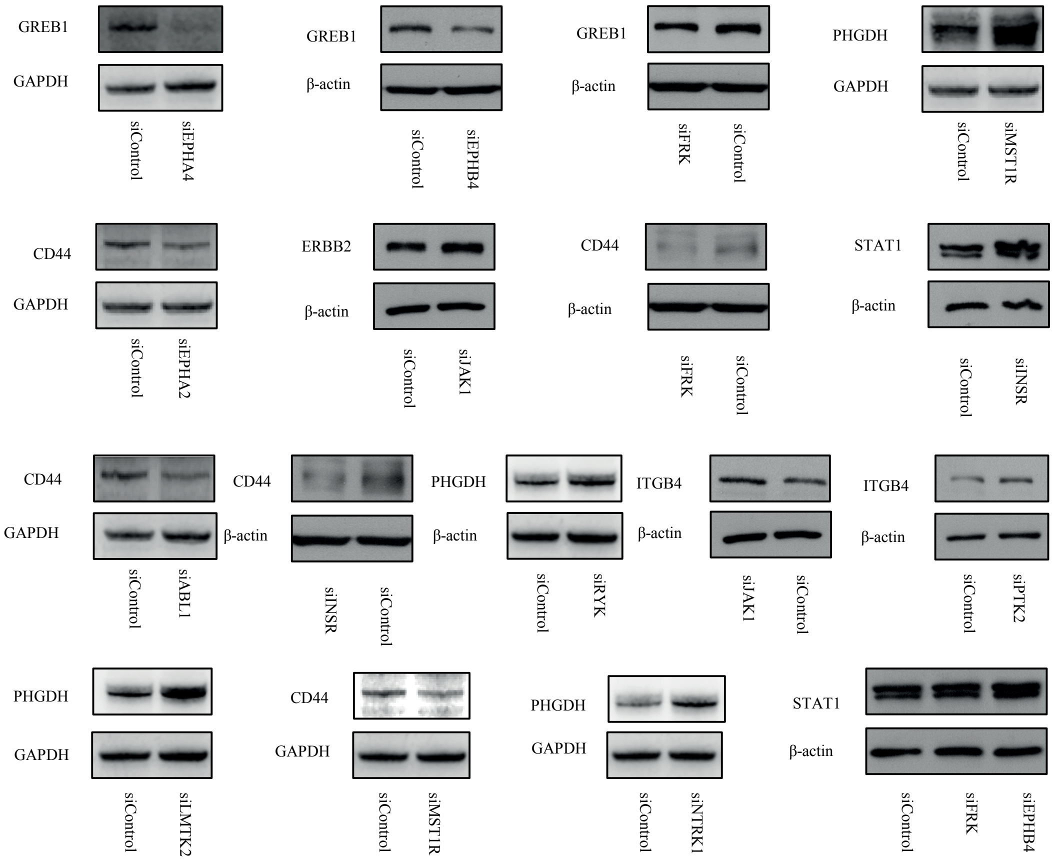

Supplemental Fig. S2. Western blotting analysis of differentially regulated proteins upon silencing of individual TKs. MCF7 cells were transfected with indicated siRNAs for 72 hours and western blotting was performed to examine the protein levels using the indicated antibodies.

Supplemental figure 3

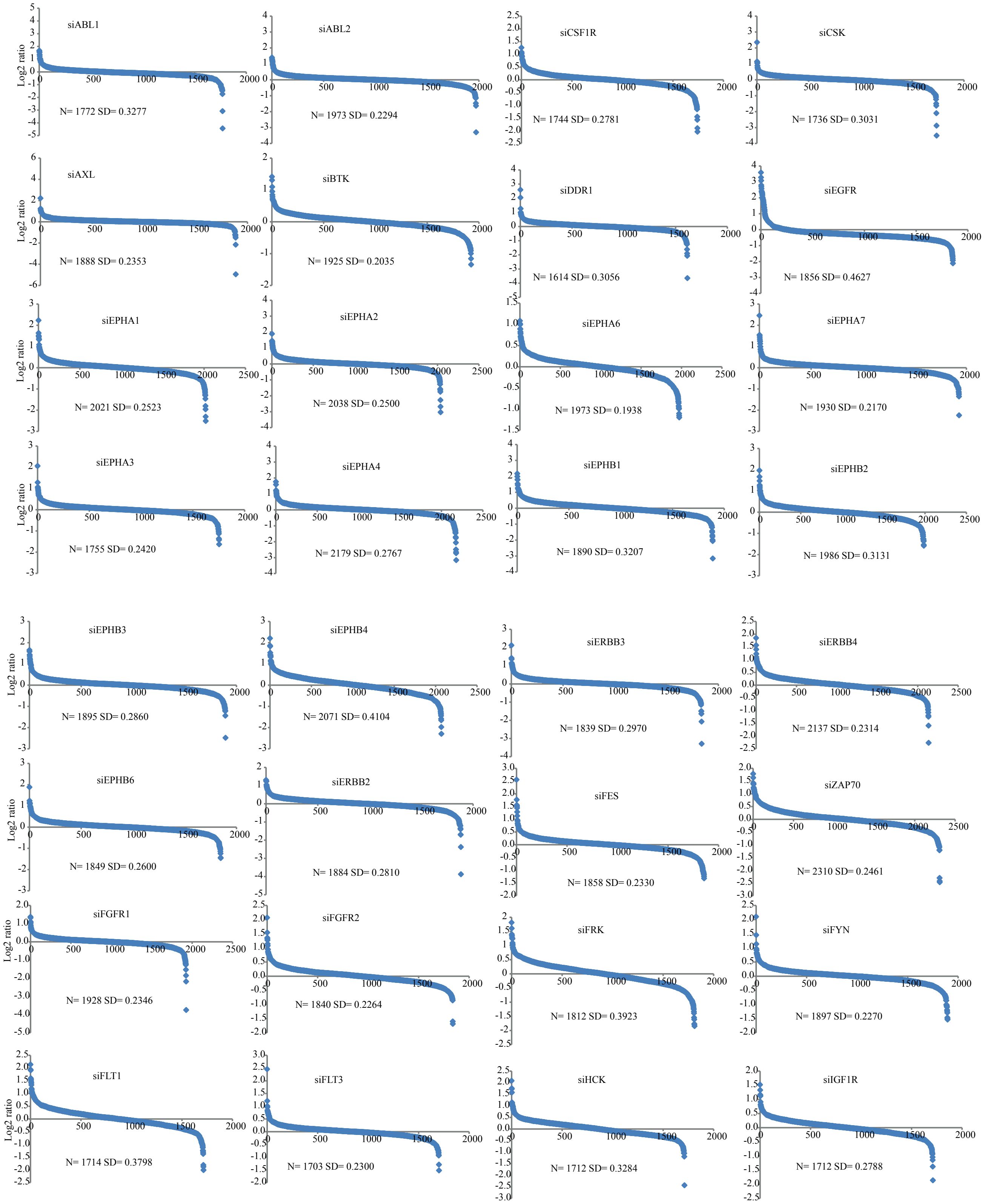

Continued supplemental figure 3

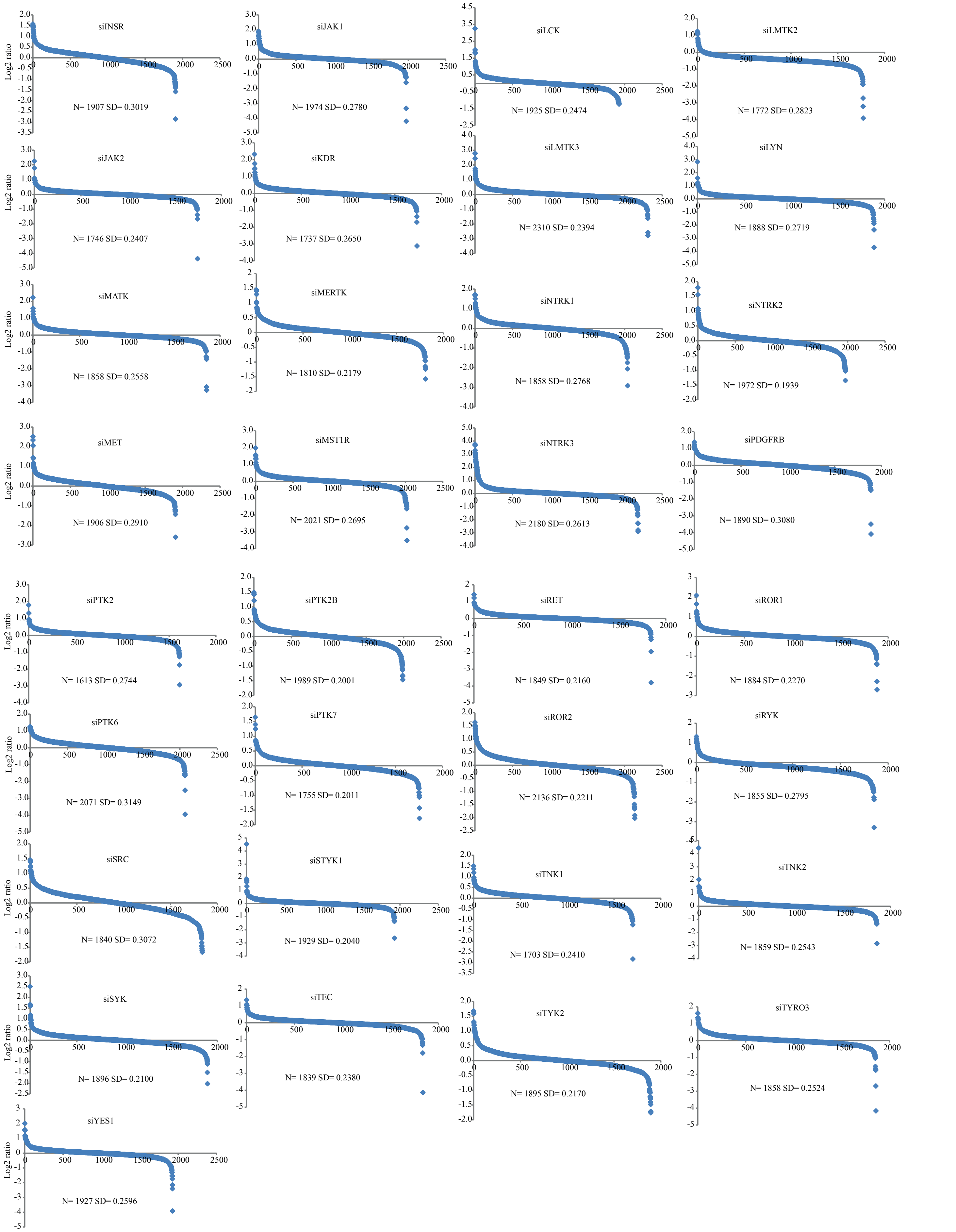

Supplemental Fig. S3. Log2 ratios distribution of siTKs vs siControl from SILAC quantitative proteomics. Distribution of Log2 normalized ratios (siTKs/siControl) of quantified proteins with a standard deviation in all 65 SILAC analyses.

Supplemental figure 4

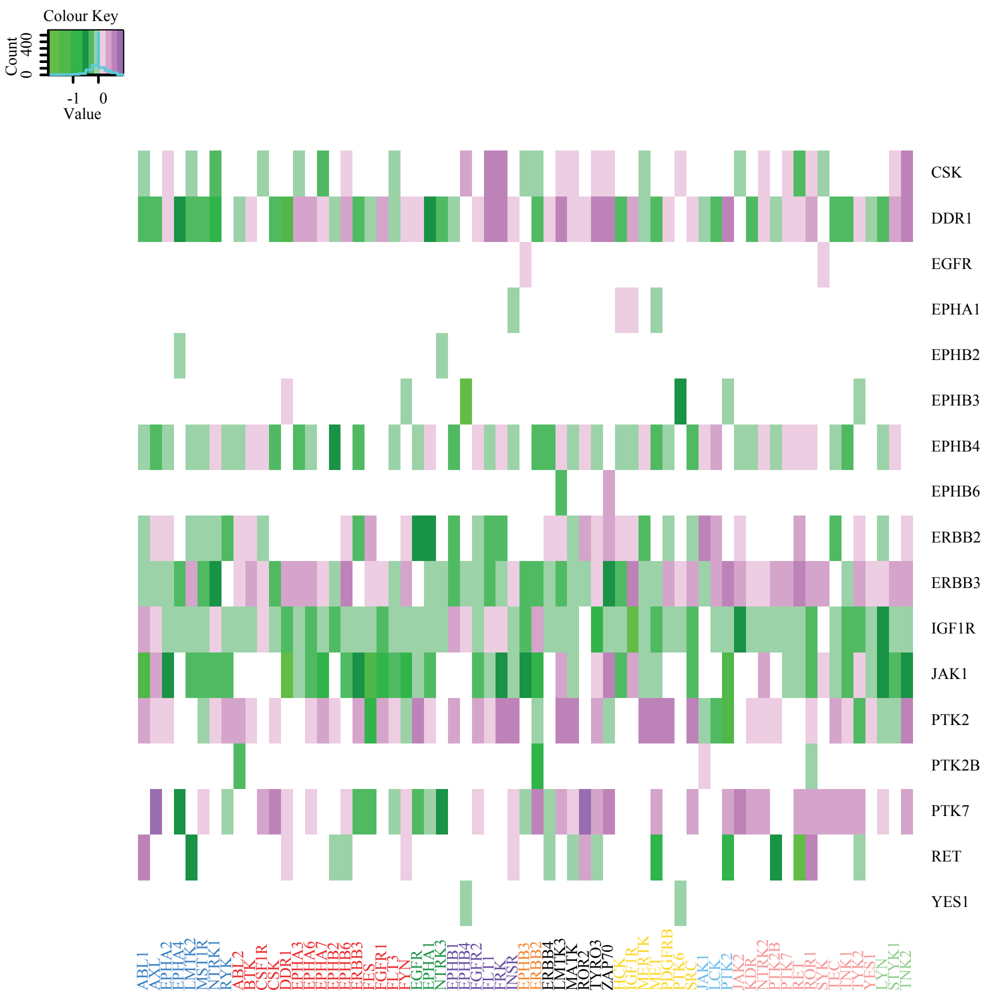

Supplemental Fig. S4. SILAC proteomic quantification of various TKs after individual knockdown of all 65 TKs. The protein expression levels of 17 members of the TK family after individual silencing of all 65 TKs were plotted in colours: green shows down-regulation and purple displays up-regulation. The white parts suggest that the silenced TK proteins were not quantified, either due to innate low abundance and technical reasons or high silencing efficiency. X-axis are the knockdowns of individual TKs, whereas y-axis are the quantified 17 TKs.

Supplemental figure 5

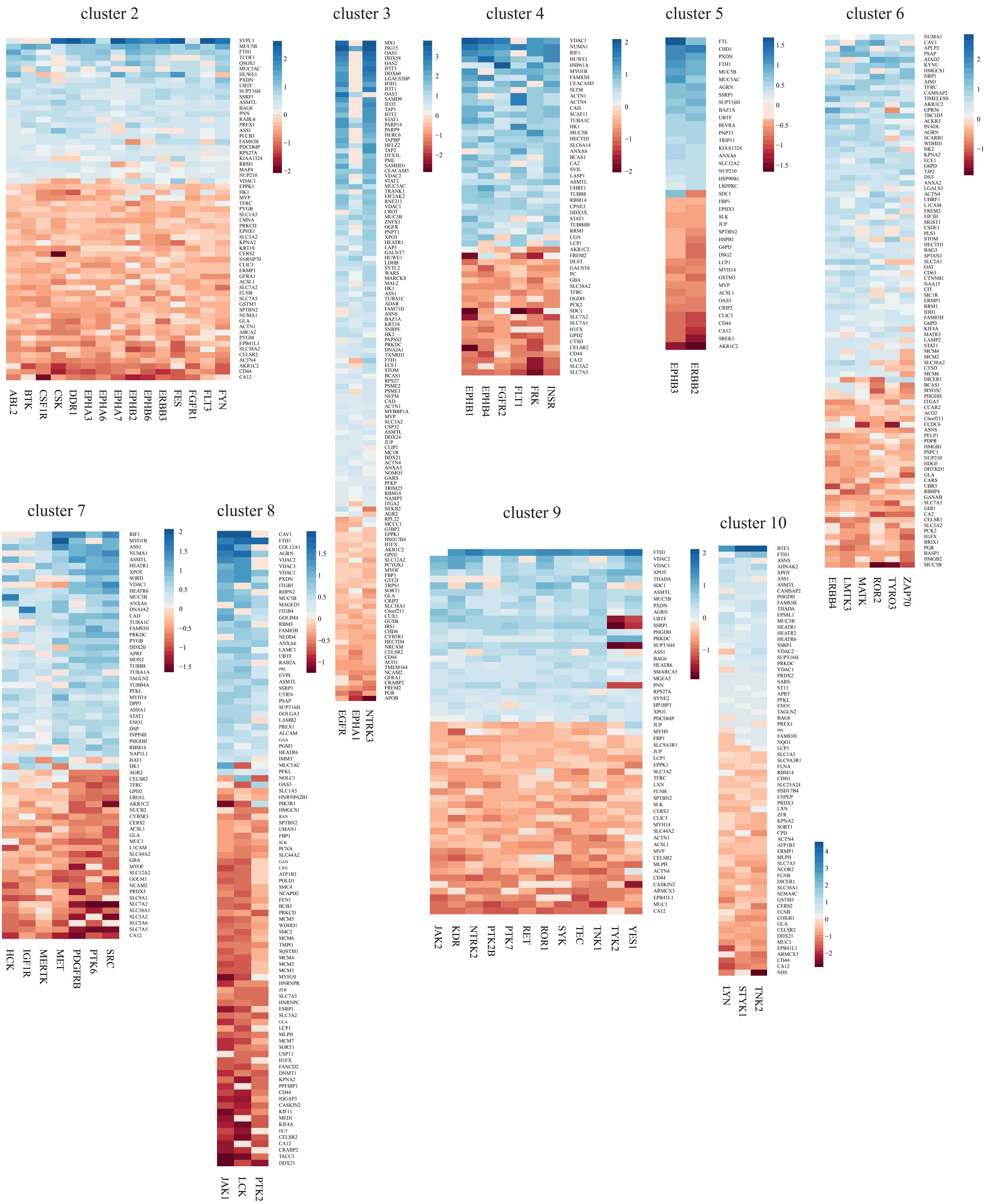

Supplemental Fig. S5. Heatmaps showing the proteomic quantifications for the downstream effects (significantly up- or down-regulated proteins) after silencing TKs in clusters 2 to 10.

Supplemental figure 6

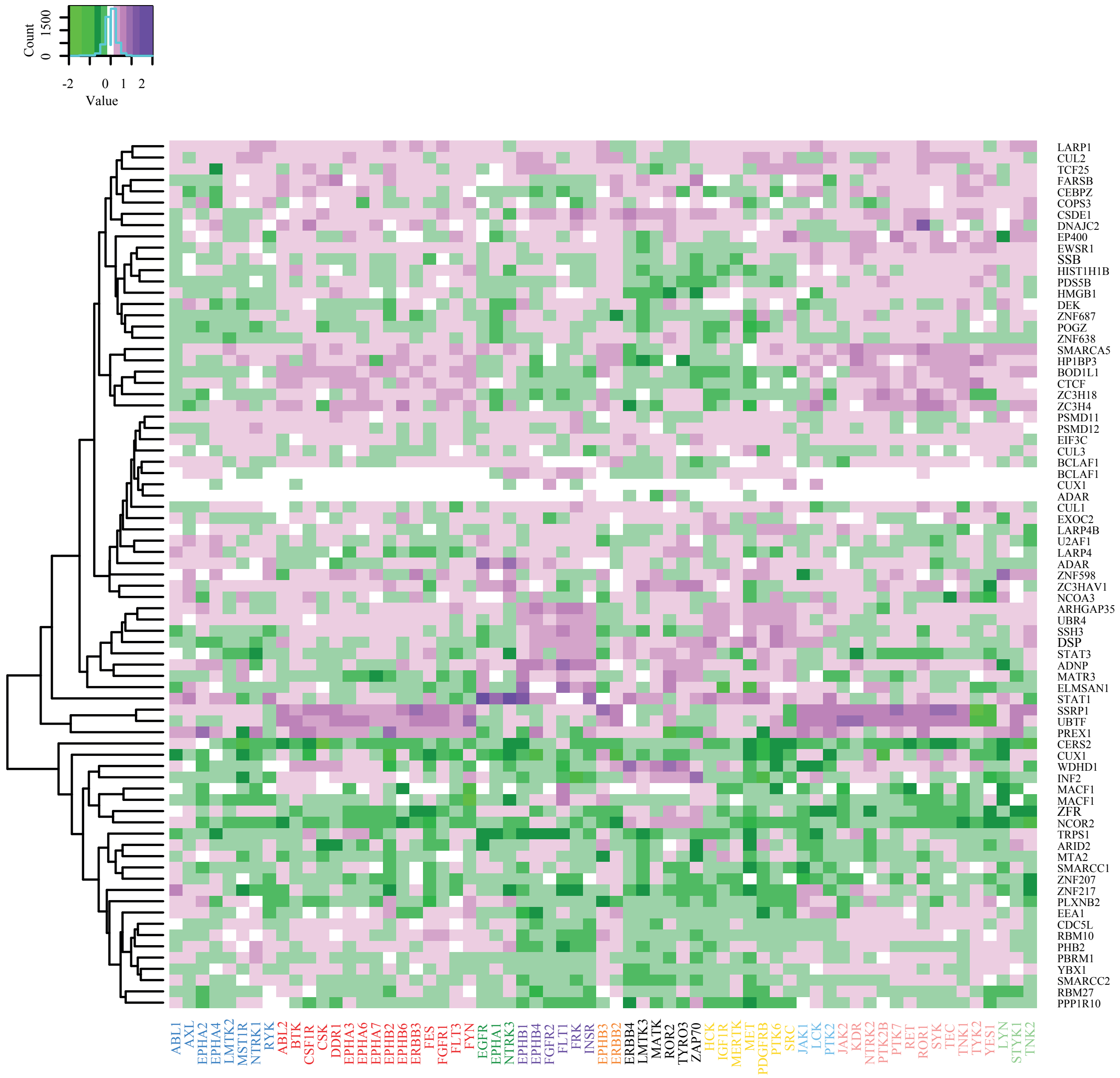

Supplemental Fig. S6. A heatmap showing the modulation pattern of 73 TFs upon individual TK knock-down. The SILAC proteomic values of 73 TFs that were identified in at least 55 knockdowns were plotted against the TKs.

Supplemental figure 7

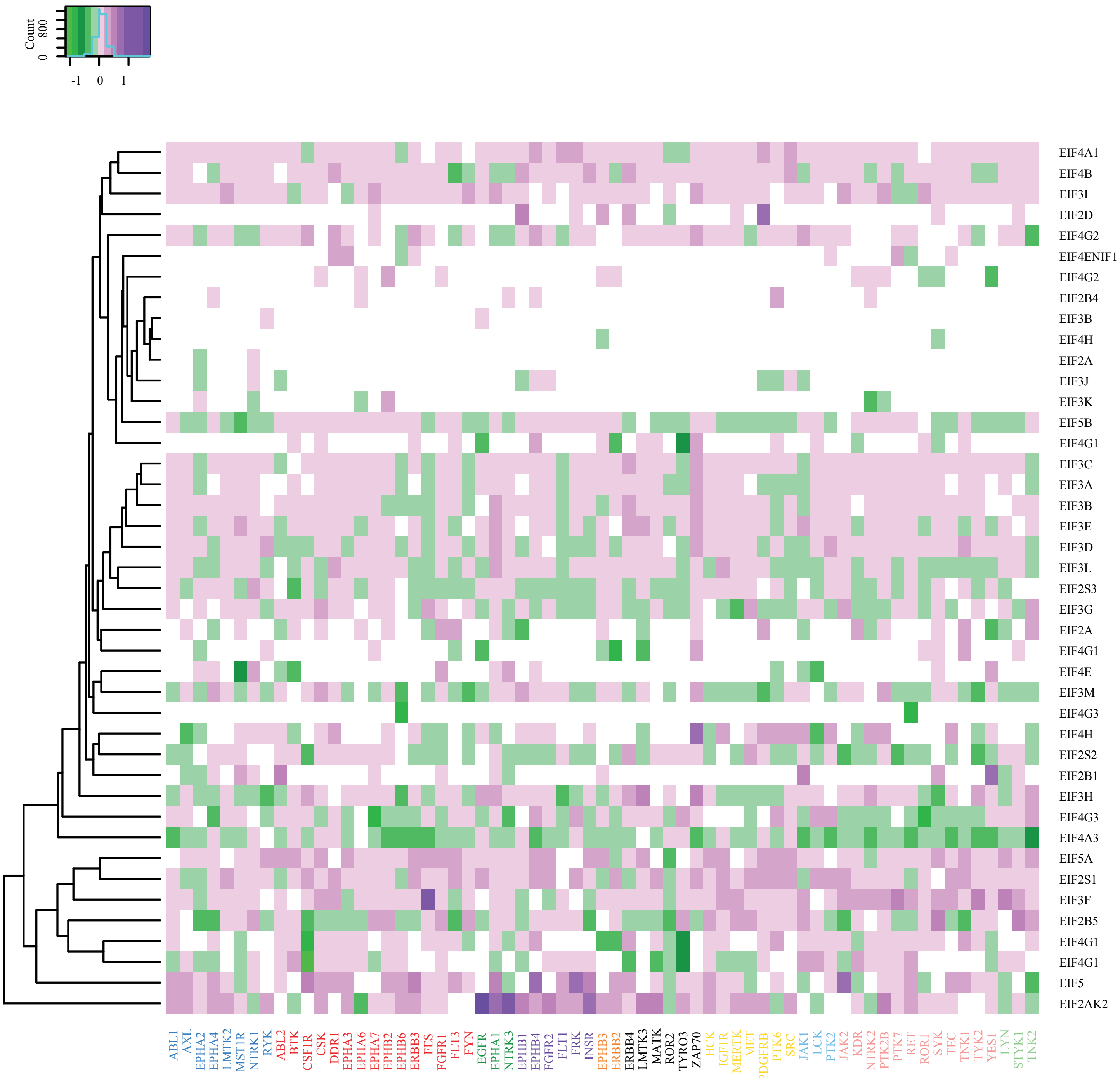

Supplemental Fig. S7. A heatmap showing the overall regulation of eukaryotic translation initiation factors (EIFs) upon individual TK knockdown. The SILAC proteomic values of 42 EIFs were plotted against the TKs.

Supplemental figure 8

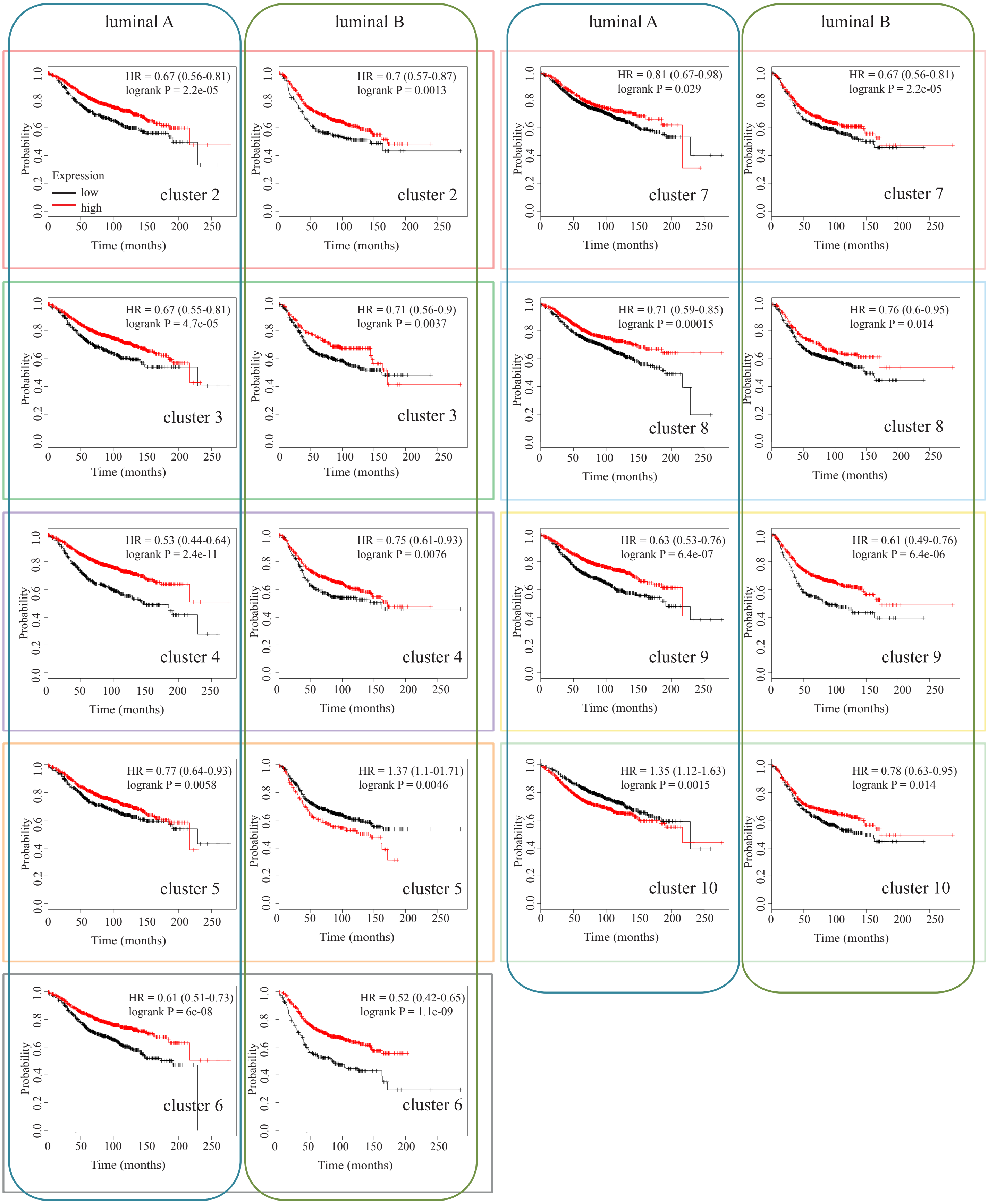

Supplemental Fig. S8. Clinical significance of each classified cluster (cluster 2 to 10) in different molecular subtypes of breast cancer. KM Plotter survival analysis, which assembles the gene expression data and survival information of more than 3,000 patients, was conducted. The mean expression levels of various TKs in each classified cluster were selected. The relevance on relapse free survival (RFS) in luminal A and luminal B breast cancer was assessed. A survival curve is displayed, and the hazard ratio (HR) with 95% confidence intervals and logrank P value were calculated and displayed.

Supplemental Table Legends

Supplemental Table 1. Full list of siRNA sequences used for targeting TKs in this study.

Supplemental Table 2. Detailed information for each identified protein by SILAC-base proteomics.

Supplemental Table 3. Key features of the SILAC-based analysis.

Supplemental Table 4. Full list of proteins that are significantly regulated in each cluster.
